# Use of body linear measurements to estimate liveweight of crossbred dairy cattle in smallholder farms in Kenya

**DOI:** 10.1186/s40064-016-1698-3

**Published:** 2016-01-22

**Authors:** M. N. Lukuyu, J. P. Gibson, D. B. Savage, A. J. Duncan, F. D. N. Mujibi, A. M. Okeyo

**Affiliations:** University of New England, Armidale, NSW 2351 Australia; International Livestock Research Institute, P.O. Box 30709, Nairobi, Kenya

**Keywords:** Body weight, Liveweight prediction, Dairy cows, Crossbreeds, Small-scale farmers

## Abstract

Body linear measurements, and specifically heart girth (HG), have been shown to be useful predictors of cattle liveweight. To test the accuracy of body linear measurements for predicting liveweight, crossbred dairy cattle of different genotypes were measured and weighed. A total of 352 mature cows and 100 heifers were weighed using an electronic weighing scale and measurements of HG, body length, height at withers were taken using an ordinary measuring tape and body condition scored (BCS) using a five-point scale. The animals were grouped according to genotype and age. Genotype classification was undertaken from farmer recall and by visual appraisal as 40–60, 61–80 or 81–100 % exotic (non-indigenous). Age classification was simply as mature cows or heifers. Liveweight of the animals ranged from 102 to 433 kg. Liveweight was strongly correlated with HG (r = 0.84) and body condition scores (r = 0.70) and moderately correlated with body length (r = 0.64) and height at withers (0.61). Regressing LW on HG measurements gave statistically significant (P < 0.01) equations with R^2^ ranging from of 0.53 to 0.78 and residual standard deviation ranging from 18.11 to 40.50 kg. The overall model developed (adjusted R^2^ = 0.71) had a prediction error of 26 kg (or 11 % of the mean) and predicted LW of over 95 % of crossbred dairy cattle in the range of 100–450 kg, regardless of age and breed group. Including BCS in the model slightly improved the model fit but not the prediction error. It was concluded that the model can be useful in making general management decisions in smallholder farms.

## Background

Liveweight (LW) forms the basis for a range of research and management activities including assessment of growth rates, responses of animals to different diets and environmental conditions and determination of feed requirements. Knowledge of animal weight and weight changes are also important in determining responses to genetic selection (Touchberry and Lush 1950), and are a key management tool (Dingwell et al. 2006; Heinrichs et al. 1992; Ozkaya and Bozkurt 2009; Touchberry and Lush 1950). The most widely accepted method globally, of measuring LW is using a calibrated electronic or mechanical scale. However, such equipment is not readily available in a smallholder farming context. Most rural farmers may also be constrained by lack of technical skills in operating and maintaining the equipment (Dingwell et al. 2006; Kashoma et al. 2011; Musa et al. 2011). Farmers and livestock traders are not accurate at estimating cattle LW, with underestimates of 46 % and overestimates of 25 % reported (Machila et al. 2008). An alternative method for estimating LW is by use of a calibrated heart girth (HG) tape. This approach was developed from measurements of Holstein heifers (Heinrichs and Hargrove 1987) and may not be applicable to the genotypes found in smallholder farms in the developing countries (Alsiddig et al. 2010), since the dairy cattle are crossbreeds of exotic breeds with different types of indigenous cattle which may differ in body structure.

Use of body linear measurements offers advantages over subjective methods of judging cattle such as visual assessment and scoring (Essien and Adesope 2003). Some authors have also suggested that it may be more reliable than weights measured with a weighing scale since the latter can be subject to short-term effects such as gut fill, urination and defecation (Russell 1975). These measurements can be taken at lower costs (when labour costs are relatively low) with a simple measuring tape and may provide relative accuracy and consistency (Guilbert and Gregory 1952; Heinrichs et al. 2007). Using 15 years of data from a Holstein herd, Touchberry and Lush (1950) analyzed repeated measurements of wither height, chest depth, body length (BL), HG and paunch girth, taken by three different people. They concluded that although the random errors of rounding reduced accuracy, a single measurement of each characteristic was accurate enough for most practical purposes provided one ensured that no gross errors occurred. Heinrichs et al. (2007) examined the repeatability of HG measurement and obtained standard deviations (SDs) of 2.19 cm among 26 observers and 2.74 cm within any one observer measuring the HG of 26 animals. The repeatability was >0.99 between multiple measurements taken by an individual observer, leading to the conclusion that the estimate of LW was highly repeatable for multiple measurements by one person or for measurements by many individuals.

Use of body linear measurements to predict LW of various types, age groups and breeds of cattle has been investigated by a number of workers. In these studies, the measurements were found to be useful in predicting weight of indigenous cattle such as Ndama of West Africa, Bali of Indonesia, working oxen in Ethiopia, Sudanese Kenana cattle, Nguni-type cattle of South Africa and the short-horn zebu cattle of Tanzania (Essien and Adesope 2003; Goe et al. 2001; Gunawan and Jakaria 2010; Kashoma et al. 2011; Musa et al. 2011; Nesamvuni et al. 2000; Sandford et al. 1982). Body linear measurements were also found to be useful in predicting weight of exotic beef cattle (Ozkaya and Bozkurt 2009; Van Marle-Köster et al. 2000) and dairy cows (Heinrichs et al. 1992; Yan et al. 2009). In Kenya, Mwacharo et al. (2006) used body linear measurements to identify and characterize two breeds of zebu cattle and found statistically significant effects of breed group, age group, sex and colour pattern on all measurements.

Numerous studies investigating the use of body linear measurements to estimate LW have focused on exotic beef, dairy and indigenous cattle. Whereas the studies concluded that these measurements can be used to obtain a reliable estimate of LW, body condition, age, breed and sex were found to influence the predictive power of the models (Kuria et al. 2007; Nesamvuni et al. 2000; Ozkaya and Bozkurt 2009; Russell 1975).

There are no LW prediction equations specific for dairy cattle in smallholder farms in Kenya. Kenya’s dairy herd is a result of many years of upgrading of the Small East African Zebu with various exotic European and American dairy breeds. It is therefore necessary to develop a prediction equation and investigate the effect of different breed compositions. The aim of this study was to test the accuracy of body linear measurements to predict LW of crossbred dairy cattle of varying exotic breeds in smallholder dairy farms in Kenya and also assess how reliable these prediction equations or those developed by others might be if used in other situations.

## Methods

This study used dairy cows and heifers owned by smallholder farmers in Siongiroi (latitude 0°55′S and longitude 35°13′E at about 1800 m above sea level) and Meteitei (latitude 00°30′N and longitude 35°17′E at about 2000 m above sea level) districts of Rift Valley Province and Kabras district in Western Province (latitudes 00°15′ and 10°N and longitudes 34°20′ and 35°E at about 1500 m above sea level).

Female crossbred cattle were weighed at selected communal dips during their normal dipping procedure where they are typically treated for cattle ticks (*Boophilus decoloratus*/*microplus*). This was done at different dip-sites over two events 15 months apart, to ensure that the same animals were not measured at the two events. The dips are normally within 3 km of the farms and hence, it is reasonable to assume that animals experienced a reasonably similar pre-weighing fast period and therefore between animal variations due to gut-fill should be negligible. As many animals as possible, were measured.

The animals were weighed to the nearest kilogram using an electronic weighing scale (ZEMIC, model H8C-C3-1.5t-4-SC) mounted on a wooden platform. The weighing scales were calibrated prior to the data collection events and again opportunistically, during data collection events. Body measurements were taken by two observers using an ordinary measuring tape and recorded in centimeters. BL was measured as the distance from the highest point of the shoulders to the pin bone; HG was measured as the body circumference immediately behind the front shoulder at the fourth ribs, posterior to the front leg. Height at withers (HW) was measured as the distance from the ground to the highest point of the withers (Brown et al. 1973; Sawanon et al. 2011; Touchberry and Lush 1950). Body condition (BCS) was scored by two observers on a scale of 1 (very poor) to 5 (fat) (DEFRA 2001; McNamara 2011; Msangi et al. 1999). A total of 352 mature cows and 100 heifers were used for the study. The animals were grouped by visual appraisal of genotype and from farmer recall as 40–60, 61–80 or 81–100 % exotic and grouped by age as mature cows (having calved) or heifers (at least 1 year old and not calved). Visual appraisal was based on the visible characteristics (such as absence of a hump, coat colour, body size) of the respective exotic compared with indigenous breed. The visual appraisal was supported with farmer recall of the breed history. From farmers’ recall of pregnancy status and visual appraisal (udder development and abdominal distension), cows that were at an advanced stage of pregnancy were excluded from the study. There were no notably serious cases of discrepancies between observers hence the full dataset was used for analysis.

### Statistical analysis

Descriptive statistics were used to present the simple means of all variables among the different breed groups. Differences between the means of the breed and age categories were compared using the least significant difference (LSD). Regression of LW on HG, BL and HW was performed using simple and multiple linear regressions with the various body measurements as continuous variables and breed as a categorical variable explaining LW. The model used was:

1 $${\text{Y}} = {\text{b}}_{0} + {\text{b}}_{1} {\text{X}}_{1} + {\text{b}}_{2} {\text{X}}_{2} + {\text{b}}_{3} {\text{X}}_{3} + {\text{e}}$$

where Y = LW, b_0_ = the intercept, X_1_ = HG, X_2_ = BL, X_2_ = HW and e = residual.

Stepwise regression using backward elimination (starting with all the predictors in the model) showed that HW had no significant effect on the model hence it was dropped. Breed group was included as a categorical variable and LW regressed on HG in a multiple regression model with groups, where breed category 1 was used as the reference. Regression equations for the different breed and age groups were compared by analysis of covariance with LW as the dependent variable, breed or age group as the factor and HG as the covariate.

The regression model developed from this study was evaluated by comparing with a number of models obtained from the literature and a commonly used calibrated weighing band (Table 1) using the mean-square prediction error (MSPE) (Yan et al. 2009). The MSPE was calculated using the following equation:

2 $${\text{MSPE}} = \frac{1}{\text{n}}\sum {({\text{P}} - {\text{A}})^{2} }$$

where P = predicted LW, A = actual LW and n = number of pairs of values being compared.

**Table 1:** Model equations from literature used for comparison with the model developed in the current study

Eqn. no.	Cattle breed/type	Equation	Source
1	Holstein heifers	LW = 100.49 − 2.830HG + 0.02636HG^2^	Heinrichs et al. (1992)
2	*Bos taurus* × *Bos indicus* crosses	LW = 4.669HG − 430.84	Msangi et al. (1999)
3	Holstein–Friesian	LW = 6.373HG − 662.6	Yan et al. (2009)
4	EA short-horn zebu	LW^0.262^ = 0.95 + 0.022HG	Lesosky et al. (2012)
5	Holstein–Friesian	LW = 5.21 HG − 473	Ozkaya and Bozkurt (2009)
6	Cross breed	LW = 7.69HG − 935	Ozkaya and Bozkurt (2009)
7	Tz short-horn zebu	LW = 4.55HG − 409	Kashoma et al. (2011)
8	*B. taurus* × *B. indicus* crosses	LW = 4.277HG − 393.13	Present study
9	Holstein heifers^a^		Common weighing band

^a^The common weighing band is based on the model developed by Heinrichs et al. (1992)

The root MSPE (residual standard deviation, RSD) $({\text{RMSPE}} = \sqrt {\text{RMSPE}} )$ and _p_MSPE (RMSPE as a proportion of mean actual LW) was used to describe the prediction accuracy (Yan et al. 2009). All the statistical analyses were carried out in GenStat 16th (VSN International 2013) with probabilities of 95 % being considered significant.

## Results

### Breed and age differences in body linear measurements

LW of cattle observed ranged from 102 to 341 kg for heifers and 152 to 433 kg for mature cows. HG measurements ranged between 107 and 188 cm, BL ranged between 81 and 136 cm and BCS from 1.5 to 4.0. All body linear measurements particularly for mature animals increased with increasing percentage of exotic genotype classifications. There was no significant difference in BCS among the breed groups except for heifers in the 61–80 % exotic breed group which had relatively better BCS (Table 2). Breed and age category influenced (P < 0.01) LW and the body linear measurements with a slight interaction effect (P = 0.03) on BW only.

**Table 2:** Mean liveweight (LW), heart girth (HG), height at withers (HW), body length (BL) and body condition score (BCS) of smallholder dairy cattle in Western Kenya

	40–60 % exotic	61–80 % exotic	>80 % exotic
LW (kg)			
Heifers	195^a^ (40.7)	212^b^ (42.6)	215^b^ (50.2)
Cows	228^a^ (39.5)	268^b^ (52.6)	325^c^ (57.7)
HG (cm)			
Heifers	141^a^ (9.6)	142^a^ (11.2)	148^b^ (13.1)
Cows	147^a^ (8.6)	153^b^ (11.2)	162^c^ (8.1)
BL (cm)			
Heifers	103^a^ (9.6)	102^a^ (9.4)	111^a^ (10.8)
Cows	105^a^ (7.2)	108^b^ (7.5)	117^c^ (6.7)
HW (cm)			
Heifers	105^a^ (2.7)	106^a^ (7.5)	113^b^ (5.0)
Cows	113^a^ (6.4)	116^ab^ (8.2)	118^b^ (5.6)
BCS			
Heifers	2.1^a^ (0.52)	2.7^b^ (0.71)	2.3^a^ (0.47)
Cows	2.7^a^ (0.71)	2.9^a^ (0.64)	2.7^a^ (0.59)

^abc^Means within a row bearing the same superscript are not different (*P* > 0.05); figures in parenthesis are standard deviation

### Correlation between body linear measurements

LW had a strong correlation with HG (r = 0.84) and BCS (r = 0.70), and a moderate correlation with BL (r = 0.64) and HW (r = 0.61). HG had moderate correlation with BL (r = 0.66) and HW (r = 0.67 and a low correlation with BCS (r = 0.57). HW had a moderate correlation with BL (0.62). LW and HG were highly correlated (r = 0.80–0.85) at all levels of body condition. Regressing LW on BCS gave a significant equation LW = 53.1BCS + 94.46 (adjusted R^2^ = 0.49; RDS = 35.66).

Correlation of rankings was significant only between BL and HW (r = 0.63), and LW and HG (0.84). Measurements of HG and BL taken by two observers were highly correlated (r = 0.91 and 0.76 respectively).

### Regression of liveweight on body linear measurements

**Fig. 1:**
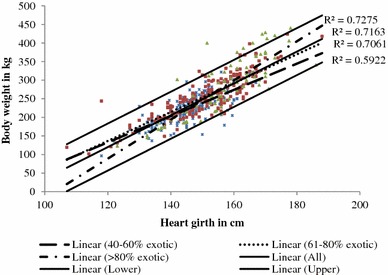
Relationship between liveweight (LW) and heart girth (HG) measurements for different breed groups, the model line (*thick solid line in the middle*) and 95 % prediction interval (*upper and lower thick solid lines*)

LW and HG had a linear relationship among all the breed categories (Fig. 1). Regressing LW on HG measurements gave statistically significant (*P* < 0.01) equations when the different breed categories were analysed separately and also when combined, with R^2^ ranging from of 0.53 to 0.78 and RSD ranging from 18 to 40 kg. The regression coefficients of the separate equations for breed and age groups were different (*P* < 0.001). When data for all the breed groups were combined, regressing LW on HG measurements gave significant prediction equation with R^2^ = 0.71 and RSD = 25.7 kg. Including BL in the model gave a significant prediction equation with R^2^ = 0.64. When genotype was included in the model as a random effect with genotype 1 as the reference, the coefficient of determination (R^2^) was 0.71. Including BCS in the model improved the model fit (R^2^ = 0.73) but not the error of prediction (RSD = 26.0 kg). Polynomial regression of LW on HG gave non-significant quadratic and cubic terms. Prediction equations of LW from HG for different breed and age groups and including BL and BCS in the model are presented in Table 3. The overall model developed using the full dataset was LW = 4.277HG − 393.13 (adjusted R^2^ = 0.705). More than 95 % of LW of the animals in the study was within the 95 % prediction interval (Fig. 1).

**Table 3:** Regression equations predicting liveweight (LW) from heart girth (HG) of cross-bred dairy cattle in smallholder farms in Western Kenya

Breed category	Regression equation	R^2^	RSD
40–60 % exotic	LW = 3.562HG − 290.44	0.59	29.36
61–80 % exotic	LW = 3.880HG − 328.94	0.72	30.98
>80 % exotic	LW = 5.257GH − 541.66	0.73	34.76
Heifers	LW = 3.4262HG − 284.33	0.76	22.78
Mature cows	LW = 4.4721HG − 419.37	0.67	33.17
Overall equation	LW = 4.277HG − 393.13	0.71	25.65
Overall with BL	LW = 3.367HG + 0.737BL − 342.6	0.65	29.73
Overall with BCS	LW = 2.76HG + 27.80BCS − 247.5	0.73	26.0
Overall equation with genotype	LW = 4.147HG + 11.78Gen2 + 11.95Gen3 − 380.9	0.71	31.9

*RSD* residual standard deviation

### Evaluation of the model

**Fig. 2:**
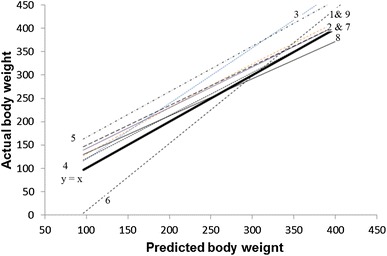
Relationship between weights predicted using models from the literature and the actual weight obtained in the present study using an electronic weighing scale (the *thick solid line* is the line of equality). Equation numbers correspond with those in Table 4

The model developed from the present study gave a prediction error of 25 kg translating to 11 % of the mean LW. When models from literature were fitted using the HG measurements from the present study, the correlation coefficients between the true and the estimated LW were >0.8 and similar to that obtained in the study (Fig. 2) but the error of prediction (RMSPE) was variable, ranging from 29 to 69 kg and proportion pRMSPE ranging from 0.119 to 0.285, which translated to 12 to 29 % of the mean LW (Table 4).

**Table 4:** Accuracy of prediction of the model developed from the present study compared with models from the literature

Eqn. no.	r	RMSPE	pRMSPE	Source
1	0.880	38.62	0.159	Heinrichs et al. (1992)
2	0.879	35.81	0.148	Msangi et al. (1999)
3	0.879	59.66	0.245	Yan et al. (2009)
4	0.876	28.86	0.119	Lesosky et al. (2012)
5	0.879	55.56	0.229	Ozkaya and Bozkurt (2009)
6	0.879	69.25	0.285	Ozkaya and Bozkurt (2009)
7	0.879	38.50	0.159	Kashoma et al. (2011)
8	0.879	25.42	0.105	Present study
9	0.874	42.5	0.17	Common weighing band

*r* correlation coefficient, *RMSPE* root mean-square prediction error, *pRMSPE* root mean-square prediction error as a proportion of the overall mean LW

## Discussion

LW and all linear measurements increased with increase in proportion of exotic genes. This was expected since the Holstein–Friesian and Ayrshire are the popular breeds used for crossbreeding by smallholder farmers in Kenya (Bebe et al. 2003; King et al. 2006; Muraguri et al. 2004). These breeds have larger body size than the indigenous animals to which they were originally crossed. However there may be an element of confounding in the data because farmers and field staff are more likely to score larger animals as having higher proportion of exotic genes because they know that exotic animals are much larger than indigenous breeds. The weights and linear measurements observed in this study were below values recorded for Holstein cattle in developed country production systems by Ozkaya and Bozkurt (2009) (LW = 513.4 kg, HG = 189.36 cm, BL = 146.37 cm, HW = 132 cm) and by Yan et al. (2009). Although the farmers in this area commonly use the Holstein–Friesian for crossbreeding, it is possible that the animals involved in the current study were at lower levels of upgrading than estimated here. On the other hand, poor nutrition has been cited as a major constraint in smallholder farms in Western Kenya (Mudavadi et al. 2001; Musalia et al. 2007; Waithaka et al. 2002) and it seems likely that the low cow sizes in the study may reflect stunted growth due to poor nutrition. The HG and HW values for the animals in this study compared (141–158 and 104–110 cm for HG and HW respectively) with those reported by Mwacharo et al. (2006) for the small East African short-horn zebu cows in Kenya (136.4–158.6 and 110–128 cm for HG and HW respectively). Being cross breeds, the animals in the present study may have been at different levels of upgrading from a type of East African Zebu smaller than the Maasai and Kamba Zebu. Despite the efforts made by the government and other organizations to introduce dairy farming, farmers in the study area were reported to keep a higher proportion of indigenous cattle due to the high cost of rearing exotic cattle (Musalia et al. 2007). The relatively lower proportion of animals in the breed group of >80 % exotic may indicate low level of dairy development in the area; hence the model developed in the present study may become an important tool to the farmers particularly when selecting dairy animals for purchase.

The LW of cows in highest exotic breed group (>80 % exotic) compared well with the weights reported from smallholder farms by Ogadi et al. (2007) for undefined Ayrshire crosses in Vihiga District (average of 300 kg) and Lanyasunya et al. (2006) (average of 325–375) under different management systems in Bahati, Nakuru District. This implies that the model developed in the present study may be useful for estimating LW of dairy animals in other parts of the country. However, where animals are of LWs above 500 kg there may be a need to validate the applicability of the model because accuracy of prediction of LW from body measurements has been found to decrease with increase in size of the animals’ body frame (Ozkaya and Bozkurt 2009). Since calves were not included in the study, it would also be important to validate the use of the model in estimating LW of calves.

Although a high SD (overall, 60 kg) for LW was observed in the present study it was similar to that obtained by other workers. Kahi et al. (2000) and Juma et al. (2006) reported SD of 70 and 45 kg respectively for LW of crossbred cattle in the Coastal Lowlands of Kenya, while Yan et al. (2009) reported a SD of 74.4 kg for Holstein–Friesian lactating cows. The high SD observed in this study may have been brought about by the variability in breed composition and age, and also variability in nutritional management across farms, which is usually associated with variation in body condition. Variability in LW may also be brought about by gut content and pregnancy (Essien and Adesope 2003; Moran 2005). This was minimized by weighing the animals very early in the morning before they were fed and avoiding animals that were evidently pregnant. Much lower SD in LW has been reported in Kenya in studies where homogeneous samples in research farms were used (Kaitho et al. 2001; Kariuki et al. 1999, 2001; Methu et al. 2001).

Whereas the SD of HG measurements (11.7 cm) obtained in the present study was higher than that obtained by Heinrichs et al. (2007) (2.19 and 2.74 cm among and within observers respectively), it was similar to that (13.8 cm) obtained by Kashoma et al. (2011) and Yan et al. (2009) (9.3 cm). Furthermore, the correlation coefficient between HG measurements by two observers was similar to that (>0.99) obtained by Heinrichs. This is an indication that HG measurements can reliably be used to predict LW even when taken by different farm mangers.

In the present study, inclusion of BL did not improve the model fit. Whereas some workers have found BL in addition to HG to improve the predictive power of the model (Gunawan and Jakaria 2010; Msangi et al. 1999; Ozkaya and Bozkurt 2009), others have not found it useful (Francis et al. 2002; Goe et al. 2001). Even in cases where improvements were found to be significant, actual reduction in the error of LW estimates was small. Since LW is most highly correlated with HG and the linear measurements are highly correlated to each other, inclusion of additional linear measurements to the prediction equation may provide little appreciable increase in accuracy of body weight estimates over equations which used HG alone (Francis et al. 2002; Goe et al. 2001). In the present study, highest correlation of LW was with HG and there was a high correlation between HG and BL hence it may not be useful to include BL in the model. This also makes the model more useful in smallholder farm conditions as it will save on the time and labour required to take additional measurement.

The highest correlation obtained in this study was between LW and HG measurement while the lowest was with HW. The high correlation between LW and HG can be attributed to the fact that, in comparison to length and HW, HG more closely reflects body condition of cows (Goe et al. 2001). This fact may also be supported by the stronger correlation observed in the present study between HG, LW and body condition scores compared to BL. Such correlations have also been reported by other workers (Francis et al. 2002; Gunawan and Jakaria 2010; Heinrichs et al. 1992, 2007; Kashoma et al. 2011; Msangi et al. 1999; Yan et al. 2009). The relationship between body linear measurements and LW could be exploited in designing appropriate management and selection programs in that high positive relationships among the traits suggests that an increase in one could lead to a corresponding increase in the other trait (Assan 2013).

Although the correlation coefficient between LW and body condition scores obtained in the present study was high (r = 0.7), the coefficient of determination (r^2^) was low. This precludes body condition score alone as a predictor of LW. Body condition score is a subjective measure which is more superior as an indicator of fat reserves and the nutritional status of dairy animals (Roche et al. 2009) and it may not be as reliable as HG measurements which is objective. Although variability in HG measurements may arise due to positioning and tension of the tape on the body of the animal, this can easily be overcome with some training and practice, which is easy to most smallholder farmers. Body condition scoring may require higher skill and practice, which may be limiting under smallholder conditions. The relationship between LW and HG measurements was high (r > 0.8) at all levels of body condition score and since body condition score has been shown to have a linear relationship with HG measurements (Nicholson and Sayers 1987), changes in body condition may well be reflected in HG measurements.

The regression equations obtained for the three breed groups when LW was regressed on HG separately were all significant. This was not surprising since the relationship between LW and body measurements has been shown to be influenced by breed, age and animal condition (Heinrichs et al. 2007; Mwacharo et al. 2006; Ozkaya and Bozkurt 2009; Taiwo et al. 2010). However, the separate equations were within the 95 % prediction interval of the overall model, which shows that the overall model can be used to estimate LW of the animals from HG measurements without stratification according to genotype or age. This has implications on the potential impact of the model as a tool which is simple and relatively accurate enough for smallholder farmers.

The model developed in the present study showed highly significant (P < 0.01) correlation between LW and HG, had a high adjusted R^2^ and predicted LW of more than 95 % of animals within 95 % prediction interval and within 11 % of the LW of animals within the range of 100–450 kg regardless of age and breed group. This weight range encompasses most animals in smallholder farms. It therefore seems reasonable to suggest that this equation is adequate when predicting LW for the purpose of making general management decisions in smallholder farms. The model developed by Lesosky et al. (2012) compared to the others used for comparison, gave LW estimates relatively similar to the model developed from the present study. This may be due to the fact that the animals used in the study were the small East African Zebu, the breed from which most of the animals in our study have been upgraded. A plot of the models however showed close relationship among animal below 250 kg, hence whether the two models can be used interchangeably to estimate LW of dairy cattle requires further investigation. The commonly used calibrated weighing band overestimated the LWs of the animals and gave a relatively high error of prediction. This may be due to the fact that it was developed from weight measurements of Holstein–Friesians in the developed countries reared under high level of management; hence it may not be appropriate for use in estimating LW of dairy cattle in smallholder farms.

Feed accounts for 50–75 % of the cost of production (Cottle 2013; Hersom 2009; Spurlock et al. 2012). It may therefore be important for smallholder farmers to monitor the nutritional requirements and efficiency of their cows for efficient utilization of available resources. In addition, feed efficiency has become an important trait in genetic selection (Spurlock et al. 2012). Whereas LW and milk production (which is less difficult to measure) are the two primary factors influencing nutritional requirements, LW drives feed intake (Hersom 2009; Prichard and Marshall undated). A change in LW of 50 kg increases the maintenance net energy (NE_m_) requirement of a cow significantly (by 6–8 %) (Lee et al. 1998; Moran 2005; NRC 2001; Prichard and Marshall Undated). With a prediction error of 26 kg, our model can be useful in estimating feed requirements and in monitoring LW changes for the purpose of matching feed to the nutritional requirements of dairy cattle. It may also be useful in measuring LW for the purpose of selecting dairy animals for body size.

When LW was regressed on body condition scores, a one-point change in BCS resulted in 53 kg change in LW. This is consistent with the findings of other workers (Enevoldsen and Kristensen 1997; Nicholson and Sayers 1987). The model may therefore be used to monitor or confirm changes in body condition. Whereas including body condition score improved the model fit, there was no improvement in the error of prediction; hence it may be more practical to use the HG measurements only under smallholder farming context. However, in monitoring the proportion of fat reserves and allocating rations for individual lactating cows where body condition is the determinant factor, body condition scoring alone may be more useful (Nicholson and Sayers 1987; Roche et al. 2009).

For the purpose of making general management decisions in smallholder farms, the general equation developed in the current study using HG measurements is applicable. This is because it provides a simple way of predicting LW to within a known prediction interval which is the overall purpose of applying the technique on-farm. From our model, a farmer would be able to estimate within 26 kg of the actual LW which is reasonable for general management decisions. This is relatively high improvement in accuracy compared to visual estimation where farmers over- or under-estimated the LW of their animals by 46.9 % (Machila et al. 2008) which translates to about 114 kg of the mean LW of the animals in the present study. A LW accuracy range of 20 % is considered acceptable for drug dosing (Machila et al. 2008) hence our model may assist smallholder farmers to make informed management decisions such as grouping animals when allocating certain rations or pastures, monitoring growth, determining LWs for breeding and market in addition to correct drug dosage. More accurate recording of live weights is rarely required in smallholder farms.

Although the regression equations for the different age and breed groups were within the 95 % prediction interval of the overall model, the accuracy of estimating LW could probably be improved by using separate equations for age and breed groups. However, age determination in this study was based on farmer recall only hence, there may be need to improve the equations for different age groups through more accurate methods of age determination such as dentition and proper records. Breed groups also, were determined based on visual appraisal of phenotypic characteristics and farmer recall and this may have resulted in bias due to subjective judgment. Accurate determination of the genotype of animals such as the ones involved in this study is confounded by the fact that use of local bulls of unknown genotype for breeding is a common practice among the farmers and hence the phenotypic characteristics may be difficult to relate to actual breed composition. A study comparing genomic and farmer prediction of breed proportions of their animals reported a correlation between the two methods of only 0.4 (Weerasinghe et al. 2013), hence the model developed from the full dataset may be more useful in predicting LW regardless of breed group. There was no need to differentiate between larger and smaller exotic breeds since it was observed from literature that farmers in that area mainly used Friesian and Ayrshire breeds for crossbreeding (Mudavadi et al. 2001; Musalia et al. 2007; Ongadi et al. 2007; Waithaka et al. 2002).

Ideally, it is important that the purpose for which LW is required is identified. For dosing purposes for instance an accuracy range of LW of 20 % is considered acceptable (Machila et al. 2008) whereas such a range may be inappropriate where animals are sold per kg LW as it may have implications on profitability of the enterprise. The magnitude of errors observed in this study is nevertheless, within the safe limits for drug.

Where a higher level of accuracy may be required for individual animals such as feed intake expressed as percent LW, daily weight gain or in the development of feed budgets for a research project, more accurate LW should be obtained by the use of electronic weighing scale. However, activities requiring such level of accuracy rarely occur in smallholder farms.

## Conclusion

The model developed in the present study predicted LW of over 95 % of crossbred dairy cattle in the range of about 100–450 kg in regardless of age and breed group, using HG measurements, which was a great improvement on visual estimation. The model can therefore be used to make important management decisions in smallholder farms such as estimating feed requirements, assessing growth rates of heifers and monitoring LW changes of dairy cows. As the variation between observers was relatively small, HG measurements will be valid even when taken by different managers. Most of the models obtained from the literature and even the commonly used weighing band overestimated LW of the animals used in the current study, suggesting there is need to validate models before applying them on different breeds, age groups and probably management systems. To achieve wider application of this method, it would be desirable to validate the equation with animals of different breed and age compositions which have been more accurately determined and under different management systems.
